# Exploring doctors’ reasons for not granting a request for euthanasia: a mixed-methods study

**DOI:** 10.3399/BJGPO.2022.0015

**Published:** 2022-10-05

**Authors:** Caroline van den Ende, Eveline Margaretha Bunge, Jennifer Eeuwijk, Suzanne van de Vathorst

**Affiliations:** 1 Pallas Health Research and Consultancy BV, Rotterdam, The Netherlands; 2 Department of General Practice/Family Medicine, Amsterdam UMC, Amsterdam, The Netherlands; 3 Department of Ethics, Law and Medical Humanities, Amsterdam UMC, Amsterdam, The Netherlands

**Keywords:** euthanasia, surveys and questionnaires, qualitative research, general practitioners

## Abstract

**Background:**

In a growing number of jurisdictions, physician-assisted death (PAD) is now an established part of medical care. Although PAD is allowed under certain criteria in The Netherlands, physicians can always refuse a request. The Euthanasia Expertise Centre (EEC) offers PAD to patients whose request was declined in circumstances where their own physician could have satisfied the legal criteria. The number of requests reaching EEC has increased, suggesting the threshold for treating physicians to refer patients to EEC has become lower.

**Aim:**

To explore the reasons of physicians for not granting a request for PAD and/or referring a patient to EEC, and any needs physicians may have in handling requests for PAD.

**Design & setting:**

Survey and interviews among Dutch physicians in The Netherlands.

**Method:**

A questionnaire was sent to 500 physicians who declined a request for PAD and whose patient subsequently requested PAD at EEC. This was followed by a qualitative study, in which in-depth interviews were held with 21 of the physicians who responded to the survey.

**Results:**

Doctors were identified as those who had objections on principle, or with other reasons for refusing a request for PAD and/or to refer the patient to EEC. These reasons were mostly related to concerns about complying with the due care criteria for PAD, or to difficulties with PAD in specific patient groups. In these cases they often valued support from another healthcare professional.

**Conclusion:**

For patients of physicians with objections on principle against PAD, EEC offered a good solution. Doctors who struggle with whether they can comply with the legal criteria might benefit from peer support.

## How this fits in

The EEC in The Netherlands offers PAD to patients whose request was declined despite it being possible for their own physician (mostly GPs) to meet the legal criteria. In a previous questionnaire study, only 3% of physicians stated that they were more likely to refuse a request for euthanasia since the founding of the EEC. The current study provides more insight into the reasons why physicians do not grant a request for PAD and/or refer a patient to EEC, and into their needs in handling requests for PAD.

## Introduction

Although in many countries the practice of PAD remains controversial, the number of jurisdictions with access to PAD is expanding, and more countries are considering legalisation.^1^ In The Netherlands, PAD is allowed under conditions (Box 1); however, physicians can always refuse to perform PAD.^2^ Physicians not willing to act on a patient's request may choose to refer their patient to another doctor, for example, a colleague, but there is no legal obligation to do so.^3^ Since 2012, there has been another option: physicians can also refer a patient to the End-of-Life Clinic.^4^

The End-of-Life Clinic was founded by Right to Die NL (*Nederlandse Vereniging voor een Vrijwillig Levenseinde* [NVVE]) to offer PAD to patients whose request was rejected despite their own physician being able to meet the legal criteria.^5^ In 2019 the name of the clinic changed to ‘Euthanasia Expertise Centre’ (EEC). The treating physician may refer the patient to EEC, but patients may also consult EEC directly. After registering, the case is assessed by a nurse and a physician working for the centre (in 2019, 140 nurses and physicians in total).^4^ If a dedicated EEC-physician is convinced, after thorough investigation, that the legal requirements can be fulfilled, they can perform euthanasia or physician-assisted suicide. However, on the principle that euthanasia should primarily be in the hands of the treating physician, EEC also offers counselling and support to physicians in order to help them in the assessment of a request for PAD.

Between 2012 and 2019, the number of requests reaching EEC increased steeply. In 2019, EEC received 3122 requests, 22% more than in 2018.^6^ In 2019, about one-quarter of the requests concerned so called ‘less complex’ requests. In general, requests from patients with terminal somatic diseases, such as cancer, heart failure, or severe lung disease, are not considered complex in The Netherlands. Another 20% concerned patients with other somatic disorders. It is expected that the number of requests will further increase (owing to the COVID-19 epidemic, the numbers of 2020 are uninformative as EEC instituted a patient stop in that year).^7^

The rise of the number of requests for PAD received by EEC, and the relatively high percentage of so-called ‘less complex’ requests handled by them, suggest that the threshold for treating physicians to refer a patient to EEC was lowered. However, little is known about the reasons treating physicians have for not granting a request for PAD themselves and/or referring a patient to the centre. In 2017, a study among 1167 physicians with specialties that regularly care for patients in the last phase of life (GPs, older care physicians, clinical specialists) showed only 3% stating that they were more likely to refuse a request for euthanasia since the founding of the End-of Life Clinic.^8^

To explore the hypothesis that the threshold for referral was lowered by having the option of EEC, the centre requested this study to investigate the reasons treating physicians have for not granting the request for PAD themselves and/or referring the patient to EEC. The secondary outcome of interest was what referring physicians’ needs are with regards handling these requests?

Box 1 **Due care criteria of the Termination of Life on Request and Assisted Suicide Act**

In The Netherlands, the Termination of Life on Request and Assisted Suicide Act, often referred to as the Euthanasia Law, (2002),^18^ allows physicians to perform euthanasia or physician-assisted suicide at a patient’s request, if they adhere to strict conditions — the so-called ‘due care’ criteria.^19^ The Act formulates the criteria of due care as follows: doctors must

1. be satisfied that the patient’s request is voluntary and well-considered;
2. be satisfied that the patient’s suffering is unbearable and without prospect of improvement;
3. have informed the patient about their situation and prognosis;
4. have come to the conclusion, together with the patient, that there is no reasonable alternative;
5. consult at least one other independent physician; and
6. perform euthanasia or assisted suicide with due medical care and attention.

Physicians also have the duty to report each completed case to the Regional Euthanasia Review Committees (RTE), who review all cases of euthanasia and assisted suicide to assess whether the physician acted in accordance with the legal criteria.^20^

## Method

### Design and population

A cross-sectional survey was conducted among physicians whose patients had requested PAD at EEC. The survey was followed by a qualitative interview study. As judged by a medical research ethics committee, the study was not subject to the Medical Research Involving Human Subjects Act. The Standards for Reporting Qualitative Research (SRQR) were used during manuscript preparation (see Appendix).^9^

### Researcher characteristics and reflexivity

CE and EB are non-medical researchers with experience in both quantitative and qualitative research in end-of-life issues. JE is a non-medical researcher with experience in qualitative data analysis in biomedical and healthcare research. SV is professor of end-of-life issues and has broad experience in this kind of research. Study set-up and every detail of execution was discussed by CE, EB, and SV. Interviews were conducted by CE. CE’s non-medical background could have been a hindrance, in that doctors may talk more freely to a colleague, but it also meant that she had to ask doctors to fully explicate. The analysis was conducted by all four authors.

### Survey

CE conducted eight exploratory interviews between May and July 2019 with physicians whose patient had submitted a request for PAD at EEC in the previous year. Based on this information CE, EB, and SV designed an online questionnaire. The questionnaire asked physicians for their experience with PAD, and for more details on the specific case. The questionnaire was sent to the first 500 physicians in 2019 whose patient applied at EEC and for whom an email address could be retrieved from the EEC files. For privacy reasons, this selection was performed by an employee of EEC. The data were obtained from 10 March until 7 May 2020. During this period a reminder email was sent out.

### Interview study

In the questionnaire, responders were also invited to participate in an in-depth interview. If they agreed to participate, they were asked to provide their personal contact details. Candidates were selected for the interviews based on the order in which the questionnaire was received (that is, first come, first served sample). Enrolment was continued until saturation was reached, that is, until three consecutive interviews yielded no new results. The interviews took place in the period from May until October 2020. The topic guide for these interviews is enclosed in the Appendix. Owing to the national measures taken to combat the COVID-19 pandemic, it was not possible to conduct the interviews face-to-face. The interviews were held by telephone or through video-conferencing and lasted approximately 1 hour. All participants gave consent for using an audio device to record the interviews.

### Data analysis

The results from the questionnaire were analysed descriptively using SPSS Statistics (version 23). All interviews were transcribed into written form and were analysed using ATLAS.ti (version 8), using grounded theory. Both CE and JE coded the interviews. After coding the first two interviews, the results were discussed before proceeding with the remaining interviews. The study used both inductive and deductive coding (part of the codes were set up and defined based on the results of the questionnaire, the other codes were built throughout the coding process). Based on the codes, common themes and sub-themes were identified. The findings were triangulated from the questionnaire and the interviews. The analysis was discussed with the supervising researchers (SV and EB).

## Results

### Characteristics of responders

A total of 116 physicians responded to the survey, which translated into a response rate of 23.2% (when taking 500 email addresses into account, including the ones that were not correct or no longer in use). Most of the responders (81.9%) were GPs. More than one-third (34.5%) was aged between 36 and 45 years, and almost all responders (94.0%) had received a request for PAD unrelated to this study (that is, other than the patient discussed in the survey). A total of 74 physicians (67.9%) had experience performing PAD (Table 1).

**Table 1.** Background characteristics of responders (*n* = 116)

Characteristic	*n* (%)
Specialism	
GP	95 (81.9)
Clinical specialist	0 (0)
Geriatric care physician	20 (17.2)
Psychiatrist	0 (0)
Junior doctor	1 (0.9)
Age	
≤35 years	19 (16.4)
36–45 years	40 (34.5)
46–55 years	23 (19.8)
≥56 years	34 (29.3)
Years of working experience	
≤5 years	9 (7.8)
6–10 years	30 (25.9)
11–15 years	22 (19.0)
>15 years	55 (47.4)
Experience with requests for PAD^a^	
Yes	109 (94.0)
No	7 (6.0)
Experience with performing PAD	
Yes	74 (67.9)
No	35 (32.1)
Number of times that PAD was performed (*n* = 74)	
1	10 (13.5)
2–5	25 (33.8)
>5	39 (52.7)

PAD = physician-assisted death. ^a^Experience with requests for PAD other than the one discussed in the survey.

Twenty-one interviews were conducted until saturation was reached: 15 with GPs, five with geriatricians, and one with a junior doctor.

### Background cases and reasons for not granting the request

Table 2 provides the main characteristics of the patient cases, namely the underlying conditions of patients, the reasons their treating physicians had for not granting the request, and any people who were consulted, as indicated in the survey. More than one-third of the cases (35.3%) concerned a patient with an accumulation of old-age complaints; 27.6% of the patients had cancer. Most physicians (79.3%) stated that they had referred the patient to EEC. Almost half of the physicians had declined the request because they doubted whether the due care criteria could be met. More than one-quarter (27.6%) mentioned other reasons, which were often related to objections against PAD in specific groups of patients, such as patients who regard their life as completed. About 17% had general objections against performing PAD. Only 2.6% stated that they believed that requests for PAD should solely be handled by EEC.

**Table 2.** Background cases and reasons for not granting the request (*n* = 116)

	*n* (%)
Underlying condition^a^	
Cancer	32 (27.6)
Other somatic disorders	21 (18.1)
Dementia	17 (14.7)
Psychiatric disorders	11 (9.5)
Accumulation of old-age complaints	41 (35.3)
Medical diagnosis not clear	9 (7.8)
Other conditions	10 (8.6)
Reasons for the treating physician not to grant the request^b^	
I have general objections against PAD	20 (17.2)
I do not want to perform PAD	16 (13.8)
I found the performance of PAD emotionally too distressing	13 (11.2)
There was not a good patient—physician relationship	15 (12.9)
I had doubts concerning the fulfilment of the criteria of due care	49 (42.2)
I was reluctant with the performance of PAD because I feared possible criminal/legal consequences	11 (9.5)
The performance of PAD should be solely in the hands of EEC	3 (2.6)
Other reasons	32 (27.6)
Has the treating physician referred the patient to a colleague?	
Yes	26 (22.4)
No	90 (77.6)
Has the treating physician contacted a consultant working for EEC?	
Yes	38 (32.8)
No	78 (67.2)
Has the treating physician consulted a SCEN-physician?	
Yes	16 (13.8)
No	100 (86.2)
Has the treating physician consulted someone else?	
Yes	36 (31.0)
No	80 (69.0)
Has the treating physician referred the patient to EEC?	
Yes	92 (79.3)
No	24 (20.7)

EEC = Euthanasia Expertise Centre. PAD = physician-assisted dying. SCEN = Support and Consultation in Euthanasia in The Netherlands. ^a^Multiple answers possible. ^b^Multiple answers possible; responders were asked to select a maximum of three answers

A total of 16 physicians (13.8%) had approached a SCEN physician (Support and Consultation on Euthanasia in the Netherlands) for support. About one-third (32.8%) had contacted a consultant from EEC, and around the same proportion (31.0%) had consulted someone else.

The diverse data did not allow the authors to do comparative analyses (that is, between certain groups of physicians).

### Needs in handling requests for PAD

Table 3 shows the main results regarding future requests for PAD. In total, 74 physicians (63.8%) considered it possible that they would be willing to perform PAD in the future; 11 physicians (9.5%) stated that they did not know. From this group of 85 physicians, 35 (41.2%) stated that they need support. More than three-quarters (77.1%) of these 35 physicians would like to receive support in dealing with requests from specific patient groups (people suffering from dementia, psychiatric disorders and people who regard their life as completed); 37.1% stated they need help with the assessment of unbearable suffering. Most (*n* = 30/35 85.7%) would like to ask one of the consultants of EEC for support.

**Table 3.** Future requests for PAD

	*n* (%)
Do you think you are willing (to continue) to perform PAD in the future?	
Yes, certainly or probably	74 (63.8)
No, certainly or probably not	31 (26.7)
Don’t know	11 (9.5)
Do you need support in handling requests for PAD? (*n* = 85)	
Yes	35 (41.2)
No	50 (58.8)
In which areas would you need support?^a^ (*n* = 35)	
Dealing with pressure from patients to grant a request	12 (34.3)
Dealing with pressure from relatives of the patient	10 (28.6)
Assessment of unbearable suffering	13 (37.1)
The legal framework for PAD	6 (17.1)
PAD in specific patient groups (dementia, completed life, psychiatric)	27 (77.1)
Other	4 (11.4)
Who would you like to ask for support?^a^ (*n* = 35)	
SCEN-physician	22 (62.9)
Consultant EEC	30 (85.7)
Colleague	19 (54.3)
Lawyer	4 (11.4)
Trainer	0 (0)
Other	1 (2.9)

EEC = Euthanasia Expertise Centre. PAD = physician-assisted dying. SCEN = Support and Consultation in Euthanasia in The Netherlands. ^a^Multiple answers possible

The results from the interviews are described below, structured by the major findings of the survey (main reasons for not granting the request, and emerging needs in handling future requests).

### Reasons for not granting the request

#### Reasons related to the fulfilment of the due care criteria

It was found that the doubts doctors had with regard to the fulfilment of the due care criteria could broadly be divided in two types. First, some doctors were not convinced that they could comply with the due care criteria. This could be because there were still treatment options, or they believed the request was not voluntary and well-considered; for example, when a patient was not consistent in expressing their wish to die. Several doctors were not convinced that the patient was suffering unbearably. Although this is a subjective experience — only the patient can determine whether their suffering is unbearable — the due care criteria demand that the physician is convinced of this unbearableness and of the lack of prospect of improvement. Some physicians found it difficult to understand, or to empathise with the suffering:

*‘Yes, we went into that case, as it were, to find out what it is that is making this lady claim that this suffering is unbearable?* […] *And I found it, each time that I was with the patient or heard from them, hard to relate to.’* (R20)

The second category concerned doubts that were more related to the possibility of legal prosecution. After all, the doctor has to comply with the due care criteria — not the patient — and risks criminal liability if they fail to do so. This makes doctors extra cautious, as was the case with the following responder:

R: *‘You know what, I do support her dying. I would have had no problem with the euthanasia due to her suffering.'*

I: *'No. But you doubted whether the due care criteria were met, and in particular whether it was actually a well-considered, consistent wish?'*

R: *'No, it’s not about that. It’s about my safety. As a healthcare professional.'*

I: *'Yes, so the fear of being prosecuted?'*

R: *'Exactly.’* (R4)

#### Reasons related to specific patient groups

Some doctors stated they had no general objections against PAD, but saw no role for themselves when it came to the performance of PAD in specific categories of patients, such as those who are ‘tired of life’:

*‘Well yes, she was … she wanted it really badly, and I can imagine to some extent that she was done with life, only there was no medical suffering so to speak. And I find that difficult. For me, that is not a reason for euthanasia.’* (R2)

Other specific groups mentioned were patients with dementia, and patients with psychiatric conditions. Although the euthanasia law does not mention ‘terminal illness’ as critical condition, several doctors stated that they had reservations about granting PAD requests for these patients. The following responder also mentioned that displaying signs of suffering is an important criterion for granting PAD in the case of dementia:

*‘I mean with someone who is terminal and that you know is going to die somewhere between now and a few months anyway, I don’t think it’s such a big problem. But if it’s someone with dementia, who could still live for years in terms of physical health and they don’t appear to be unhappy at all, yes, I find that a difficult case, yes.’* (R3)

Furthermore, patients with dementia or psychiatric disorders might lack insight into their disease, and therefore are unable to satisfy the requirement that their request be both voluntary and well-considered. Some patients with dementia write, when they are still mentally competent, an advance directive requesting PAD in specific circumstances. The following responder considered the fact that the doctor is not able verify whether this desire still exists at the time of PAD, as a ‘no go’:

*‘*[…] *but as a doctor I can’t test whether you still want that.* […] *That is my opinion, my personal opinion, you have to be able to test it. And if you can no longer test it because the patient has become mentally incapacitated, then that’s too bad.’* (R20)

With regard to PAD for patients with a (suspected) psychiatric disorder, several physicians expressed concerns regarding the often uncertain prognosis of such a condition, and the options there might still be for treatment:

*‘I thought maybe there is a lot more going on psychiatrically and there is actually a lot of therapy that hasn’t been tried yet.’* (R10)

#### Reasons related to general objections against PAD

Physicians with categorical objections against performing PAD stated this was because of a (Christian) religious belief or a certain philosophical view leading them to not want to decide over life or death, or to intervene in the process of dying. Some of the doctors with general objections against performing PAD stated that not wanting to trouble a colleague with a request for PAD made them decide to refer to EEC, because the workload is already high, and/or because the number of requests for PAD is rising. Also, as this physician stated, it may not always feel ‘safe’ to refer to a colleague:

*‘Because then you have okay, fine, my colleague has to go there for a talk. They will of course also ask “how are things going?” And I feel a little judged on my treatment plan, so to speak. Yes, to me that feels a bit like someone is snooping around in my case.’* (R8)

### Needs in handling requests

When physicians’ needs in handling requests for PAD were explored further, several physicians stated that they appreciate the opportunity to discuss the case with other healthcare professionals. They emphasised the added value of a ‘sparring partner’, for example, when it concerns a psychiatric case:

*‘Two psychiatrists from the End-of-Life Clinic, they came to visit with the Balint group.* […] *Yes that really gave me a lot of support at the time, that you also know that you can indeed call someone if you run into an issue *[…]*’* (R21)

The following responder valued consulting a professional when establishing the (subjective) concept of unbearable suffering:

*‘Well, I notice that when I look back at the euthanasia cases that I have struggled with, that it’s often about that, about the unbearable part.* […] *But sometimes it’s nice to be able to ask a real outsider, “hey, what do you think about this?”’* (R14)

Another responder stated that it is helpful to have a colleague present during the performance of PAD:

*‘What I often do in practice is to take a colleague with me, but that also feels like a burden.’* (R7)

A different category of needs concerned broader needs such as informing society (for example by GPs or the media) about other humane ways of dying, as a response to the changing attitudes of the general public towards having more control over one’s choice to live or die. Some physicians expressed the concern that people increasingly consider PAD as a right, and as something that can easily be arranged:

*‘And as easily as the question can be put to you these days, that’s actually pretty special.’* (R6)

Other physicians suggested changing the practice of PAD, that is, a review procedure before PAD, instead of an assessment after performing PAD. This would give physicians more certainty that they act according to the legal criteria, as this responder argued:

*‘And in terms of legislation, well you see right … what I find very unfortunate about the euthanasia law is that it always assessed afterwards.’* (R12)

## Discussion

### Summary

This mixed-methods study explored the reasons that treating physicians had for not granting a request for PAD and/or referring the patient to EEC. Physicians were also asked whether they had any unmet needs for handling these requests. The study found that doctors who did not grant PAD and/or referred a patient to EEC can be categorised into 1) doctors with (mostly religion-based) objections to performing PAD in principle, and 2) doctors with other reasons. These other reasons were often related to concerns about fulfilling the due care criteria. Other physicians had difficulties with PAD in specific patient groups, such as patients with dementia, psychiatric disorders, and those with accumulation of old-age complaints. The study does not support the hypothesis that having EEC available made doctors decline a request for PAD more easily.

### Comparison with existing literature

The findings are in line with a previous study that showed that only a small proportion of doctors were more likely to refuse a request for PAD since the founding of EEC.^8^ This study also showed that a minority of doctors is willing to grant PAD in complex cases, especially when it concerns patients with a completed life.

The ‘right’ to die for those with a completed life has been the subject of public debate for a number of years and remains a topic of discussion in The Netherlands. Recently, the state was sued by a private organisation (Coöperatie Laatste Wil) that wants to make it possible for Dutch citizens who regard their life complete to end their lives in a 'humane way'.^10^ Some physicians in the present study expressed their concerns over this growing emphasis on patient autonomy in ending one’s life. Paradoxically, current developments in society that place more emphasis on PAD as a patient choice seem to make physicians more reluctant when it comes to granting or performing PAD. This holds true in particular for patients with age-related conditions, often involving non-medical problems such as loneliness, mourning, a loss of meaning, or dependence on care.^11^

Other than for patients with a completed life, the suffering of patients with an accumulation of old-age problems has a medical basis (mostly degenerative disorders, for example visual or hearing impairment, cognitive decline, or joint pain) and is therefore allowed under the Dutch law. More than one-third of the cases in the present study concerned such a patient with an accumulation of old-age complaints. A doctor from EEC suggested in a journal by the Royal Dutch Medical Association (RDMA) that these doctors mistake these patients for those with a completed life, and therefore refer them too quickly to EEC.^12^

Some of the physicians in the current study mentioned that they appreciated support from another healthcare professional when handling a request for PAD, especially when they had concerns regarding the fulfilment of the due care criteria (in complex cases). It was found that this support can be offered in various ways, for example in the form of a Balint group^13^ or other ways of interprofessional support. For doctors who fear criminal prosecution, support will probably not change that. The responders preferred a review procedure *before* PAD instead of *after,* as currently regulated by the law, and such reassurance-seeking may indicate that they still fear criminal prosecution. This is remarkable, since in the 20 years that the Dutch Euthanasia Act has been in effect now, only one doctor has been brought to criminal court, and very few cases were judged as not complying with all criteria: in 2020 there were 6938 notifications of PAD, and in only two cases the RTE found that the physicians did not act in accordance with the due care criteria.^14^

Interestingly, although some of the physicians in the study were looking for support, approaching a SCEN physician for this purpose did not seem a good option to them. SCEN physicians, who are often consulted as the independent physician to assess whether the due care criteria are met, should also be able to provide support (as indicated by the ‘S’ in SCEN) and advise on any legal or ethical issues the treating doctor might have.^15^ Unlike consultants of EEC, they cannot offer long-term support though, and their support may threaten their independence, thus complicating the procedure.^16^

### Strengths and limitations

A strength of the study is the use of quantitative and qualitative methods (triangulation), with which an extensive view was gained of the reasons of physicians to decline a request for PAD and/or to refer a patient to EEC. It is acknowledged that the study also has limitations. For the survey, cases were selected on the basis of the availability of an email address, and the group for whom an email address could be retrieved may not be representative for all treating physicians. Second, the low response rate of the survey (23.2%) might have resulted in selection bias, if treating physicians who refer a patient more easily to EEC were less likely to fill out the questionnaire. Third, the interviews in the qualitative part of the study could not be held face-to-face owing to national measures against COVID-19. The absence of visual or non-verbal cues is often seen as a drawback of telephone interviews.^17^ The responders were offered the possibility of video-conferencing to try and overcome this issue. The advantage of the ‘first come first served’ sampling approach is that selection occurred without any relation to the content of the questionnaire answers; the possible disadvantage is that the early responders were selected, which may lead to a bias towards doctors motivated to participate.

### Implications for practice

In conclusion, it was found that some of the doctors who did not grant PAD and/or referred a patient to EEC had objections on principle. Others reasons for refusal or referral mostly related to concerns about being able to comply with the due care criteria. For the first group with objections on principle against PAD, referral to the EEC offered a good solution. Doctors in the second group would value support in assessing the patient and interpreting the due care criteria. Unfortunately, there currently seems to be a lack in support systems, and those that exist, such as the services EEC offers, are largely unknown.

When other jurisdictions make PAD possible, the authors recommend that they consider that physicians who have to deal with PAD requests would benefit from a good support structure, and this would also indirectly benefit the patients. Moreover, in such a new situation the physicians will not have had any education or training in how to deal with PAD requests so additional training facilities will also be much needed.

## Data

The dataset relied on in this article is available from the corresponding author on reasonable request.
